# Benchmarking of survival outcomes following haematopoietic stem cell transplantation: A review of existing processes and the introduction of an international system from the European Society for Blood and Marrow Transplantation (EBMT) and the Joint Accreditation Committee of ISCT and EBMT (JACIE)

**DOI:** 10.1038/s41409-019-0718-7

**Published:** 2019-10-21

**Authors:** John A. Snowden, Riccardo Saccardi, Kim Orchard, Per Ljungman, Rafael F. Duarte, Myriam Labopin, Eoin McGrath, Nigel Brook, Carmen Ruiz de Elvira, Debra Gordon, Hélène A. Poirel, Francis Ayuk, Yves Beguin, Francesca Bonifazi, Alois Gratwohl, Noel Milpied, John Moore, Jakob Passweg, J. Douglas Rizzo, Stephen R. Spellman, Jorge Sierra, Carlos Solano, Fermin Sanchez-Guijo, Nina Worel, Andreu Gusi, Gillian Adams, Theodor Balan, Helen Baldomero, Gilles Macq, Evelyne Marry, Florence Mesnil, Elena Oldani, Rachel Pearce, Julia Perry, Nicole Raus, Urs Schanz, Steven Tran, Leonie Wilcox, Grzegorz W. Basak, Christian Chabannon, Selim Corbacioglu, Harry Dolstra, Jürgen Kuball, Mohamad Mohty, Arjan Lankester, Sylvia Montoto, Arnon Nagler, Jan Styczynski, Ibrahim Yakoub-Agha, Regis Peffault de Latour, Nicolaus Kroeger, Ronald Brand, Liesbeth C. de Wreede, Erik van Zwet, Hein Putter

**Affiliations:** 1 Department of Haematology, Sheffield Teaching Hospitals NHS Foundation Trust, Sheffield, UK; 2 Haematology Department, Careggi University Hospital, Florence, Italy; 3 Department of Haematology, Southampton General Hospital, Southampton, UK; 4 Department of Cellular Therapy and Allogeneic Stem Cell Transplantation, Karolinska University Hospital, Division of Hematology, Department of Medicine Huddinge, Karolinska Institutet, Stockholm, Sweden; 5 Servicio de Hematologia y Hemoterapia, Hospital Universitario Puerta de Hierro Majadahonda, Madrid, Spain; 6 EBMT Study Office, Hôpital Saint Antoine, Paris, France; 7 EBMT Executive Office, Barcelona, Spain; 8 EBMT Registry Office, London, UK; 9 Belgian Cancer Registry, Brussels, Belgium; 10 Department of Stem Cell Transplantation, University Medical Center Hamburg, Hamburg, Germany; 11 Department of Haematology, CHU and University of Liège, Liège, Belgium; 12 Institute of Hematology “Seràgnoli”, S. Orsola-Malpighi University Hospital, Bologna, Italy; 13 Department of Hematology, Medical Faculty, University of Basel, Basel, Switzerland; 14 CHU Bordeaux, service d’hematologie et therapie Cellulaire, F-33000 Bordeaux, France; 15 Department of Haematology, St Vincent’s Hospital Sydney, Darlinghurst, NSW Australia; 16 EBMT Activity Survey office and Swiss National Transplant Registry SBST, Basel University Hospital, Switzerland, Basel, Switzerland; 17 Center for International Blood and Marrow Transplant Research, Milwaukee, WI USA; 18 Center for International Blood and Marrow Transplant Research, Minneapolis, MN USA; 19 Hematology Department, Hospital Sant Pau, Barcelona, Spain; 20 Servicio de Hematología, Hospital Clínico de Valencia, Valencia, Spain; 21 IBSAL, Hospital Universitario de Salamanca, Salamanca, Spain; 22 Blood Group Serology and Transfusion Medicine, Medical University of Vienna, Vienna, Austria; 23 Department of Biomedical Data Sciences, Leiden University Medical Center, Leiden, The Netherlands; 24 Agence de la biomédecine, Paris, France; 25 Italian National BMT Registry (GITMO), Bergamo, Italy; 26 BSBMT Data Registry, London, UK; 27 Société Francophone de Greffe de Moelle et de Thérapie Cellulaire (SFGM-TC) Centre Hospitalier Lyon Sud, Pierre-Bénite, France; 28 Haematology, University Hospital, Zurich, Switzerland; 29 Australasian Bone Marrow Transplant Recipient Registry (ABMTRR), Darlinghurst, NSW Australia; 30 Department of Hematology, Oncology and Internal Medicine, Medical University of Warsaw, Warsaw, Poland; 31 Institut Paoli Calmettes & Centre d’Investigations Cliniques en Biothérapies, Marseille, France; 32 Department of Pediatric Hematology, Oncology and Stem Cell Transplantation, University of Regensburg, Regensburg, Germany; 33 Department of Laboratory Medicine, Radboud University-Nijmegen Medical Centre, Nijmegen, The Netherlands; 34 Department of Haematology, University Medical Center Utrecht, Utrecht, The Netherlands; 35 Service d’Hematologie Clinique, Saint-Antoine Hospital, AP-HP, Sorbonne University, and INSERM UMRs 938, Paris, France; 36 BMT Centre, Leiden University Hospital, Leiden, The Netherlands; 37 St. Bartholomew’s and The Royal London NHS Trust, London, UK; 38 Chaim Sheva Medical Center, Tel-Hashomer, Israel; 39 Collegium Medicum, Nicolaus Copernicus University, Bydgoszcz, Poland; 40 CHU de Lille, Université de Lille, Lille, France; 41 Saint Louis Hospital, Paris Diderot University, Paris, France

**Keywords:** Haematological cancer, Haematopoietic stem cells

## Abstract

In many healthcare settings, benchmarking for complex procedures has become a mandatory requirement by competent authorities, regulators, payers and patients to assure clinical performance, cost-effectiveness and safe care of patients. In several countries inside and outside Europe, benchmarking systems have been established for haematopoietic stem cell transplantation (HSCT), but access is not universal. As benchmarking is now integrated into the FACT-JACIE standards, the EBMT and JACIE established a Clinical Outcomes Group (COG) to develop and introduce a universal system accessible across EBMT members. Established systems from seven European countries (United Kingdom, Italy, Belgium, France, Germany, Spain, Switzerland), USA and Australia were appraised, revealing similarities in process, but wide variations in selection criteria and statistical methods. In tandem, the COG developed the first phase of a bespoke risk-adapted international benchmarking model for one-year survival following allogeneic and autologous HSCT based on current capabilities within the EBMT registry core dataset. Data completeness, which has a critical impact on validity of centre comparisons, is also assessed. Ongoing development will include further scientific validation of the model, incorporation of further variables (when appropriate) alongside implementation of systems for clinically meaningful interpretation and governance aiming to maximise acceptance to centres, clinicians, payers and patients across EBMT.

## Introduction, aims and objectives

The complexity of modern medicine and demands in healthcare for accountability, transparency, cost-effectiveness and quality improvement requires definition of quality of care. Stakeholders, including specialist and referring clinicians, public health bodies, patients and families wish to understand the differences in survival and other patient outcomes across centres. Benchmarking, defined as “the process of comparing a practice’s performance with an external standard” (https://www.ahrq.gov/professionals/prevention-chronic-care/improve/system/pfhandbook/mod7.html. Accessed 31/05/2019), aims to provide a means of comparing clinical performance with the wider dataset. Benchmarking has become mandatory in a number of countries for a range of medical and surgical procedures and is used by competent authorities, regulators and payers for quality assurance of clinical effectiveness, safety and cost-effectiveness of patient care [1–7]. However, there are challenges in the delivery of benchmarking, particularly in defining a fair, accurate and clinically meaningful methodology acceptable to all stakeholders underpinned by a reliable and complete source of clinical outcomes data. For long-term sustainability, there is also a need for evidence of sufficient impact on patient outcomes to justify the considerable time and resources invested [7].

Haematopoietic stem cell transplantation (HSCT) is a high-risk treatment which requires a multi-disciplinary approach and often complex interactions with different teams and services [8]. Risks are dependent on the type of transplant procedure, and also patient-related and disease-related factors, and patients may be consented for levels of treatment-related mortality risk from around 1% to higher than 30%, and balanced against the outcomes with other non-transplant treatments [8–13]. The procedure-related risks are highest in the first year but frequently persist for several years following transplant, reflecting the profound immune suppression and other chronic complications associated with some HSCT procedures. Relapse is also a feature of the post-transplant pathway and, to a degree, reflects the patient selection and transplant technique [13].

External quality review of HSCT centres through accreditation by FACT-JACIE standards has become established over the last two decades and its adoption is associated with improved survival outcomes, especially in allogeneic HSCT [14, 15]. Benchmarking has been integrated into the FACT-JACIE quality management standards since the 6th edition in 2015 [16], which included the standard B4.7.5: *The Clinical Programme should achieve one-year survival outcome within or above the expected range when compared to national or international outcome data* (FACT-JACIE International Standards for Hematopoietic Cellular Therapy Product Collection, Processing and Administration, 6th edition March 2015). The 7th edition, published in 2018, has developed this further under standard B4.7.6 mandating benchmarks for non-relapse mortality at 100 days (FACT-JACIE International Standards for Hematopoietic Cellular Therapy Product Collection, Processing and Administration, 7th edition March 2018).

In several countries inside and outside Europe benchmarking systems for HSCT outcomes have been established [17, 18]. However, the extent and methodologies of benchmarking across EBMT national members have not previously been critically appraised. Access to a nationally based benchmarking system is not universal across EBMT and it may be unfeasible, even meaningless, in small countries with limited centres and populations.

Therefore, there is a significant unmet need for a consistent system, open to all EBMT member centres, to benchmark survival outcomes against national and/or international norms. Given the impact of pre-transplant variables on outcome of HSCT, risk status needs to be adequately incorporated into benchmarking methodology [18–21], otherwise individual centres, or even national HSCT communities, may be reluctant to accept the validity of results, particularly if they indicate relative centre (or national) underperformance. Importantly, there should also be no deterrent to treating high-risk patients based on concerns that overall centre performance may be affected. Systems will be essential to advising centres appropriately how to address and correct underperformance.

The upgrading of the EBMT registry, now with over 660,000 HSCT registrations, from the ProMISe system to the new MACRO platform, has provided an opportunity by which survival outcomes benchmarking could be addressed across the EBMT membership. The development of benchmarking was therefore integrated as a ‘work package’ within the broader ‘Project 2020’ registry upgrade via Clinical Outcomes Group (COG). The ‘work package’ scoped existing national benchmarking systems, both within and outside of EBMT, to inform the development of statistical methodology for a universally available risk-adapted benchmarking system, to be ultimately operated within the new MACRO-based registry and overseen by JACIE. This review summarises the first stage (or ‘first phase’) in a longer-term development of a fair, acceptable and sustainably delivered international benchmarking system across the diverse health services and cultures within EBMT.

## Methods

**Fig. 1:**
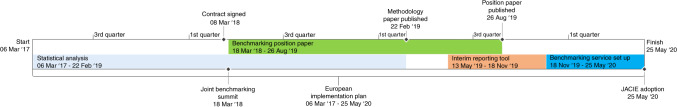
Timeline for projected development of clinical outcomes benchmarking process and scoping of established benchmarking systems

As an intrinsic part of the EBMT registry upgrade ‘Project 2020’, a work package *“Clinical quality assurance of patient survival outcomes by international benchmarking”* was incorporated. Following initial discussions within meetings of the EBMT Board and EBMT Annual Meeting in Marseille in 2017–18, the Leiden University Medical Centre (LUMC) team was appointed to develop statistical methodology for benchmarking for the EBMT. Subsequently, a multi-national group of senior HSCT clinicians, registry managers, EBMT (including JACIE) staff and biostatisticians from LUMC, EBMT Patient Advocacy Committee and national societies met systematically as the ‘Clinical Outcomes Group’ on a monthly basis from the Lisbon Annual Meeting in March 2018–June 2019. CIBMTR and Australasian (ABMTRR) representatives were co-opted in order to access their experience. After agreeing Terms of Reference, discussions progressed either by e-mail or teleconference with periodic in-person meetings (see Fig. 1). Statistical aspects were led by the Department of Biomedical Data Sciences, LUMC with input from the EBMT Statistical Committee and national statistical representatives. Data was sourced from the EBMT registry which is supported by mandatory reporting of anonymised data from routinely consented patients by full EBMT centre members, and supplemented with data from the broader EBMT activity survey. The introductory phase of the benchmarking system and plans for development were finalised and ratified at the EBMT Board meeting in June 2019.

National HSCT organisations providing existing benchmarking systems were identified and representatives were approached to join the extended membership of the Clinical Outcomes Group and contribute information that characterised the individual approaches to benchmarking. A survey was sent out to the representatives to characterise their processes, which are summarised in Tables 1–3. Also, information about how results are presented to the participating centres, and examples of plots and tables were requested.

**Table 1:** Summary and comparison of patient selection criteria used in established benchmarking systems and in the first phase of EBMT benchmarking system (final column)

Subject attribute	BSBMT —UK	GITMO—Italy	SBSCT—Switzerland	DRST—Germany	BTR—Belgium	ABM—France	GETH —Spain	ABMTRR-Australia/New Zealand	CIBMTR—USA	Phase 1 EBMT
Year benchmarking commenced	2010	2012	1997	1998	2012	2004	2017	2002	1994	2019
Type of HSCT benchmarked: Allo/Auto	Yes/Yes	Yes/No	Yes/Yes	Yes/Yes	Yes/Yes	Yes/No	Yes/Yes	Yes/Yes	Yes/No	Yes/Yes
Transplant Number, e.g, 1st, all	All	All	1st	1st	All	1st	All	1st	1st	1st
No. of transplant years included in analysis cohort	6	From 2012	20	10	7	5	4	5	3	5
Centre Inclusion criteria used? Yes/No	No		Yes	Yes	Yes	Yes	Yes	No	Yes	Yes
Centre inclusion criteria if used e.g. minimum number of transplants or outcome events	N/A	All transplants irrespective of number of HSCT per centre	>20	>20 “events”	≥5/year	≥5/year	EBMT members	N/A	NONE— unstable estimates for less than 10 events	REFER TO TEXT

**Table 2:** Summary and comparison of outcome measures used in established benchmarking systems and in the first phase of EBMT benchmarking system (final column)

Outcome measure	BSBMT—UK	GITMO—Italy	SBSCT— Switzerland	DRST—Germany	BTR—Belgium	ABM—France	GETH —Spain	ABMTRR—Australia/New Zealand	CIBMTR—USA	Phase 1 EBMT
1-year overall survival	Yes	Yes	Yes	Yes	Yes	Yes	Yes	Yes	Yes	Yes
3-year overall survival	Yes	No	Yes	No	Yes	No	No	Yes	No	No
5-year overall Survival	Yes	No	Yes	Yes	No	No	No	No	No	No
10-year overall survival	No	No	Yes	No	No	No	No	No	No	No
1 year non-relapse mortality (NRM)/transplant-related mortality (TRM)^a^	Yes	Yes	Yes	Yes	Yes	Yes	Yes	Yes	No	No
Relapse incidence at 1 year	Yes	Yes	Yes	Yes	No	Yes	Yes	Yes	No	No
Completeness of follow-up—are you reporting completeness of follow-up to reporting centres? Yes/No	Yes	Yes	Yes	Yes	Yes	No	Yes	Yes	Yes	Yes

^a^See JACIE definition

**Table 3:** Summary and comparison of covariates used in established benchmarking systems and in the first phase of EBMT benchmarking system (final column)

Covariate	BSBMT—UK	GITMO—Italy	SBSCT—Switzerland	DRST—Germany	BTR—Belgium	ABM—France	GETH—Spain	ABMTRR—Australia/ New Zealand	CIBMTR—USA	Phase 1 EBMT
Transplant year	No	Yes	Yes	Yes	Yes	Yes	Yes	No	Yes	Yes
Recipient age	Yes	Yes	Yes	Yes	Yes	Yes	Yes	Yes	Yes	Yes
Recipient sex	Yes	Yes	Yes	Yes	Yes	Yes (donor match)	Yes	Yes	Yes	Yes
Recipient race	Yes	No	No	No	No	No	No	No	Yes	No
Recipient CMV status	Yes	Yes	Yes	Yes	Yes	Yes (donor match)	Yes	No	Yes	Yes
Previous autologous HSCT	No	Not excluded; all allogeneic HSCT independently of previous autologous HSCT	Yes	No	No (not as such but as overall transplant number)	Yes	Yes	No	Yes	Yes
Type and status of disease	Yes	Yes	Yes	Yes	Yes	Yes	Yes	Yes	Yes	Yes
Time from diagnosis	No	Yes	Yes	Yes	Yes	No	Yes	Yes	Yes	Yes
Conditioning	Yes	Yes	Yes	Yes	Yes	Yes	Yes	MA/RIC	Yes	Yes
Stem cell source	Yes	yes	Yes	Yes	Yes	Yes	Yes	Yes	Yes	No
Donor type	Yes	Yes	Yes	Yes	Yes	Yes	Yes	Yes	Yes	Yes
Donor age	No	No	Yes	Yes	Yes	Yes	Yes	No	Yes	Yes
Donor sex	No	Yes (mismatch)	Yes	Yes	Yes (as donor sex match)	Yes	Yes	Yes	Yes	Yes
Donor CMV match	Yes	Yes	Yes	Yes	Yes	Yes	Ye**s**	No	Yes	No
Karnofsky/Lansky	Yes	Yes	Yes	Yes	Yes	No	Yes	No	Yes	Yes
HLA matching	Yes	No	Yes	yes	Yes	Yes	Yes	Yes	Yes	No
Disease risk index (Armand)	No	No	Yes	No	Yes	No	Yes	Yes	N/A	Yes
Disease risk index (Armand) with cytogenetics	No	No	Yes	No	Yes	No	No	No	N/A	Yes
EBMT Risk score	No	No	Yes	Yes, age-adjusted	No	No	No	Yes	N/A	Yes
Comorbidity/HCT-CI	Yes	No	Yes	No	No (available only in some centres)	No	Yes	No	Yes	Yes
Comments	Although recorded, the covariates are not used for risk adjusted analysis		Calculated from data reported via the newest version of the EBMT Registry Minimal Essential Data MED-A form	Although recorded, the covariates are not used for risk adjusted analysis					Casemix only applied to allogeneic HSCT. 3 years survival reported for autologous HSCT only.	Adjusts for disease risk using disease status and other clinical, cytogenetic and molecular factors

Tables 1–3 summarise and compare the different approaches. To accompany the tabulated information, summaries of each national approach to benchmarking are provided in the Supplementary Information part 1. The processes, membership and roles within the Clinical Outcomes Group are summarised in Supplementary Information part 2.

### Development of a ‘first phase’ statistical model for benchmarking of 1-year survival across EBMT centres

The initial work focused on constructing case-mix-corrected funnel plots, a well-known tool for performance evaluation, to compare centres to the national or European average performance with respect to 1-year overall survival. The benchmarking mechanisms considered a series of risk factors (“case mix” variables) to be integrated into the statistical models to allow for a fair comparison of centres related to different patient population characteristics. The output was to be a risk-adjusted comparison of each centre with the internal benchmark, set by the average across participating EBMT centres.

As part of the early development work, the LUMC methods were applied to data from British and Italian centres for comparison with the existing BSBMT and GITMO systems. These two national systems were identified in initial scoping as being examples of well-established and accepted national systems within EBMT, albeit with some weaknesses such as lack of risk adaptation. Six other national systems were subsequently used to inform the modelling process. Members of the CIBMTR were co-opted into the group to relay the experience of the well-established CIBMTR benchmarking system for allogeneic HSCT. The goal was not to supplant existing performance evaluations but to supplement them and to facilitate comparisons across Europe and inform the development of a model that could be accepted and used by EBMT centres, with uptake and roll out driven by JACIE accreditation requirements.

One major issue recognised at an early stage was the completeness of data within the existing EBMT data. Experience from other benchmarking systems combined with examinations of the EBMT database in recent years highlighted the fundamental importance of high data completeness. Both follow-up and variables that were considered clinically relevant to risk adjustment needed to be complete. With respect to the latter, although there was a desire among the clinical members to incorporate modern genetic prognostic data and a consistently reported degree of HLA-matching data into the adjustments, it became apparent that these and other variables were not reliably reported in the current database and therefore would have to be considered for future reporting via the mandatory core dataset. Thus, to an extent, the selection of the variables for ‘first phase’ was based on an appraisal of the available data and subsequent consensus across the group.

### Implementation and resourcing

The group discussed model methods and assumptions, interpretation and limitations of the results, and considered the impact of the introduction of a benchmarking system with a process to be opened in which EBMT would decide how the benchmarking information should be delivered to the centres and how centres should be supported in their interpretation of results. Formal consultation with the EBMT membership has been ongoing throughout the developments to enable amendments before finalisation. This included several updates via EBMT Newsletters and a Special Session dedicated to Benchmarking (Special Session dedicated to Benchmarking. https://www.ebmt.org/ebmt/news/plenary-and-special-sessions-ebmt-2019. Accessed 08/07/2019) at the Annual EBMT Congress in Frankfurt in March 2019.

In tandem, the EBMT Board, Registry Committee and JACIE Committees serially reviewed the programme at their regular meetings and allocated resources for initial development of modelling, testing and roll out with a view to sustainable delivery via the JACIE Office.

### Selection of cases

The EBMT benchmarking project targets only first autologous HSCT and first allogeneic HSCT (including those preceded by an autologous transplant), which cover around 80% of all transplant activity, because inclusion of subsequent transplants complicates calculation of survival post-transplant. In addition, for both allogeneic and autologous HSCT, patients treated for solid tumours were excluded. For autologous HSCT, only transplants for adults with haematological cancers (lymphoma, myeloma, and acute leukaemia) were included, with exclusion of paediatric transplants (recipient <18 years old) and other rare indications, such as autoimmune diseases, which will be addressed at a later stage of development.

### Selection of centres for benchmarking

Inclusion criteria for centres included:

- Full membership of EBMT;
- During the 4-year period 2013–2016 at least 80% of the transplants reported in the Activity Survey for the centre were registered in the EBMT;

Additional inclusion criteria for centres for benchmarking allogeneic and autologous transplants were defined as follows based on the JACIE minimum activity thresholds:

- Allogeneic transplants: during the period 2013–2016 a minimum of 10 allografts per year on average;
- Autologous transplants: during the period 2013–2016 a minimum of 5 autografts per year on average

Selections were made separately for allogeneic and autologous transplants. The 4-year time frame for selection of centres was dictated by the availability of separately collected activity reports. This data was used to compare actual registrations with expected levels of activity as reported by the centres. Future benchmarking efforts will use a 5-year period for selection of centres.

### Selection of covariates for case mix correction

The statistical goal was to develop a model comparing the performance of all EBMT centres against a benchmark; this benchmark is set internally by determining the performance across all centres by means of a regression model. Using such a model, it is possible to adjust for relevant patient, disease and transplant characteristics known to affect HSCT outcomes. This protects centres treating difficult patients from appearing to provide sub-standard care, where poor performance may be due to the unfavourable case mix.

The CIBMTR had previously determined a list of relevant patient, disease and transplant characteristics and this list was also incorporated, to the extent that the suggested variables are available within the EBMT registry. Thus, the variables that describe the case mix of allogeneic HSCT to be used by EBMT were defined as follows:

- Diagnosis (and disease status/stage)
- Donor type: matched sibling donor vs. other related vs. unrelated donor
- Coexisting disease (HCT-specific comorbidity index, HCT-CI)
- Recipient age
- Donor age
- Recipient and donor gender
- Recipient cytomegalovirus (CMV) serological status
- Recipient Karnofsky/Lansky Performance Status score at transplant
- Prior autologous transplant
- Time from diagnosis to transplant for acute myelogenous leukaemia (AML) and acute lymphoblastic leukaemia (ALL) not in CR1 or primary induction failure (PIF) (used as surrogate for length of CR)
- Year of transplant

The same variables are used for autologous HSCT, except coexisting disease, interval diagnosis—transplant, recipient cytomegalovirus (CMV) serology, CR1 vs. not CR1, prior autologous transplant (and age of donor, donor type and biological donor gender).

### Incorporating diagnosis (at transplant), disease status/stage (at transplant): disease risk index (DRI)

Armand et al developed a DRI [19] with four categories; Low, Intermediate, High and Very high. The EBMT have validated DRI in EBMT data for allogeneic HSCT [20] and the DRI has been examined in more specific contexts.

For our ‘first phase’ model, we started with a ‘crude’ version of the DRI which does not take cytogenetics into account, using the exact definition of Armand et al. [19]. The DRI categories including cytogenetics were subsequently obtained from the EBMT registry (Myriam Labopin, personal communication), using the categorisation in Table 4, and merged into the file for the ‘first phase’ statistical model, enabling a cross-tabulation of the DRI without cytogenetics versus the DRI with cytogenetics. All missing cytogenetic data for MDS and AML patients were coded as “intermediate cytogenetic risk” [19]. This led to a re-classification of DRIs, especially for autologous transplants, with many of the DRI’s that were classified as intermediate being reclassified as low. Diseases not included in the original DRI were categorised based on similar diseases; all non-malignant diseases were considered as low risk. For a future benchmarking ‘second phase’ an improved refined DRI considering more detailed cytogenetic information is to be implemented by the Leiden statistical team.

**Table 4:** Refinement of the disease risk index (DRI, derived from Armand et al, reference 19) as applied to the first phase of the EBMT benchmarking system

Disease	Stage	DRI group
Hodgkin lymphoma CR		Low
CLL CR		Low
Mantle cell lymphoma CR		Low
Indolent NHL CR		Low
AML favourable cytogenetics CR		Low
Indolent NHL PR		Low
CLL PR		Low
CML chronic phase 1/2		Low
CML advanced phase		Int
Mantle cell lymphoma PR		Int
Myeloproliferative neoplasm	Any	Int
AML intermediate cytogenetics CR		Int
ALL CR1		Int
T-cell NHL CR		Int
Multiple myeloma CR/VGPR/PR		Int
Aggressive NHL CR		Int
Low-risk MDS adverse cytogenetics	Early^†^	Int
T-cell NHL PR		Int
Low-risk MDS intermediate cytogenetics	Early^†^	Int
Hodgkin lymphoma PR		Int
Low-risk MDS intermediate cytogenetics	Advanced^†^	Int
Indolent NHL	Advanced^†^	Int
CLL	Advanced	Int
High-risk MDS intermediate cytogenetics	Early	Int
Aggressive NHL PR		Int
T-cell NHL	Advanced^†^	High
AML favourable cytogenetics	Advanced^†^	High
Hodgkin lymphoma	Advanced^†^	High
High-risk MDS intermediate cytogenetics	Advanced^†^	High
High-risk MDS adverse cytogenetics	Early	High
ALL CR2		High
AML adverse cytogenetics CR		High
Mantle cell lymphoma	Advanced^†^	High
High-risk MDS adverse cytogenetics	Advanced^†^	High
Burkitt lymphoma CR		High
Multiple myeloma	Advanced^†^	High
ALL CR3		High
Low-risk MDS adverse cytogenetics	Advanced^†^	High
AML intermediate cytogenetics	Advanced	High
CML blast phase		Very high
ALL	Advanced^†^	Very high
Aggressive NHL	Advanced^†^	Very high
AML adverse cytogenetics	Advanced^†^	Very high
Burkitt lymphoma PR	Advanced^†^	Very high

^†^Advanced stage refers to induction failure or active relapse, including stable or progressive disease for NHL, HL or CLL

Although a substantial number of absent variables remained, an expert consensus emerged on the need to reflect the clinical importance of cytogenetics despite limited available data. Therefore, a decision was taken to develop a ‘default’ system within the ‘first phase’ model i.e. applying the ‘crude’ DRI with cytogenetics from the DRI with cytogenetics where available from the EBMT registry file, but when missing replaced by the ‘crude’ DRI without cytogenetics.

### Outcome and data quality

To be able to make a reliable comparison of one-year survival across centres, it is essential to have adequate follow-up. Unfortunately, the quality of follow-up differs substantially between centres. It was therefore decided to firstly benchmark all centres on the quality of their follow-up. That is, loss to follow-up within one year after HSCT is the outcome of interest; death is censoring the follow-up length. This approach is similar to the use of the reverse Kaplan–Meier for estimating the follow-up distribution.

As loss to follow-up could hamper the benchmarking on mortality, it was therefore decided to fit benchmark models for mortality using only the 50% centres with the best quality of follow-up. The decision which centres have the best follow-up is based on the *p*-values associated with benchmarking follow-up.

### Missing case mix data

Missing case mix data was dealt with differently when fitting the benchmark models and when performing the actual benchmarking. For fitting the benchmark models, multiple imputation was used to avoid any bias due to missingness at random. However, when doing the actual benchmarking, missing case mix variables are imputed by their median/mode among all patients within the EBMT with observed favourable outcome (for benchmarking one-year mortality this means patients that survived one year after HSCT).

This will make the patient appear “relatively healthy”, and lead to an unfavourable observed over expected ratio for the centre. This should encourage centres to strive for complete registration of case mix variables.

### Benchmarking with funnel plots

The benchmark regression model can perform the following tasks:

- Compute the expected number of unfavourable outcomes within each centre;
- Compare this expected number to the number of unfavourable outcomes observed;
- Perform a statistical test of the hypothesis that a centre’s performance does not differ from the benchmark;
- Quantify the evidence for under- or over-performance of each centre by means of the p-value of that test;
- Represent the results graphically in a so-called funnel plot [22]. The performance of centres that fall outside of the funnel may be considered to be significantly different from the benchmark at a fixed level of significance, usually 5%.

The funnel plot has become a standard tool for centre comparisons and is visually attractive and easy to interpret. In most applications, funnel plots are based on binary outcomes. However, when benchmarking one-year mortality the funnel plot needs to be adjusted to time-to-event data that may be subject to censoring. While the same principle applies, there is a need to use Cox regression instead of logistic regression to construct the benchmark. The full technical details of our approach are beyond the scope of this position paper and fully described in “Statistical use of Cox method Funnel Plots in clinical benchmarking HSCT” (van Zwet et al., unpublished).

### Benchmarking one-year mortality

For benchmarking one-year mortality, the expected number of unfavourable outcomes within a centre is obtained from calculating the probability of observing a death before one year for each patient in the centre, under the assumption that the survival distribution after case mix correction is the same for all centres (this is the null-hypothesis) while the censoring distribution may differ between centres. If *T* is the event time (death) and *C* is the censoring time (length of follow-up in case of no event), then the probability of observing a death for a particular patient in the centre within one-year equals (time is in months)

$$\begin{array}{l}P(T \, < \, C,T \, < \, 12{\mathrm{|}}{\mathrm{{centre,patient}}}\;{\mathrm{{characteristics}}})\\ \hskip 55pt = \mathop {\int }\limits_{t = 0}^{12} P\left( {T = t{\mathrm{|}}{\mathrm{{patient}}}\;{\mathrm{{characteristics}}}} \right)P\left( {C \, > \, t{\mathrm{|}}{\mathrm{{centre}}}} \right){\mathrm{{d}}}t.\end{array}$$

To compute this probability, we use a case mix-corrected model for the distribution of *T* and a centre-specific model for the distribution of *C*.

### Benchmarking one-year loss to follow-up

For benchmarking one-year loss to follow-up, we change the event of interest from “dead before one year” to “lost to follow-up within one year”. So, for each patient we compute the probability of observing the censoring event before one year under the assumption that the censoring distribution is the same for all centres (this is the null hypothesis), while the survival distribution may differ between centres. To be precise, the probability of observing the censoring event before one-year equals

$$\begin{array}{l}P(C \, < \, T,C \, < \, 12{\mathrm{|}}{\mathrm{{centre}}},{\mathrm{{patient}}}\;{\mathrm{{characteristics}}})\\ \hskip 65pt = \mathop {\int }\limits_{t = 0}^{12} P\left( {C = c} \right)P\left( {T \, > \, c{\mathrm{|}}{\mathrm{{centre,patient}}}\;{\mathrm{{characteristics}}}} \right){\mathrm{{d}}}c.\end{array}$$

To compute this probability, we use an uncorrected model for the distribution of *C* and a centre-specific, case mix-corrected model for the distribution of *T*.

In addition to visualising one-year loss to follow-up in a funnel plot, we also quantified the completeness of follow-up using the methods of Clark et al. [23] namely by calculating for each centre the total follow-up time $\mathop {\sum }\nolimits_j t_{ij}$, divided by the total maximum follow-up time $\mathop {\sum }\nolimits_j t_{ij}^ \ast$. The maximum follow-up time $t_{ij}^ \ast$ in this case is either the time of death, if the patient dies within one year, or one year, if the patient is alive at the end of one year [19]. Only patients transplanted in 2013–2016 were included in this calculation. The centres are categorised into one of three categories, with colour coding, intended to be used as an additional signal, next to the funnel plot:

1. Centre has very good follow-up (>90%) fulfilling proposed EBMT criteria and can be included in the benchmarking: (GREEN—compliant);
2. Centre has a follow-up of borderline completeness (80–90%) provisionally fulfilling proposed EBMT criteria and can be included this time but needs to improve to stay in the system: (AMBER—partially compliant);
3. Centre has a reported follow-up of a quality not allowing benchmarking (<80%) and are therefore below proposed EBMT criteria which could affect JACIE accreditation: (RED—noncompliant)

## Results

### Benchmarking for allogeneic HSCT

A total of 288 centres, providing data for 61,826 patients have contributed to this benchmarking project for allogeneic transplants.

### One-year mortality

**Fig. 2:**
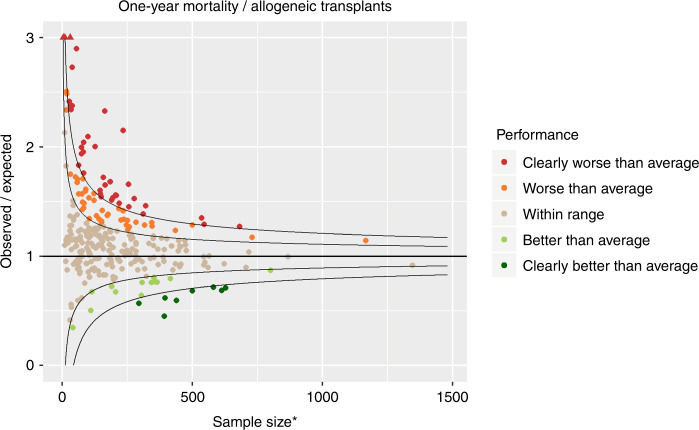
Funnel plot for 1-year mortality following allogeneic transplantation comparing observed over expected mortality adjusted for case mix and centre follow-up. Results are highly affected by quality of follow-up over the period. *Adjusted for case mix and centre follow up

Figure 2 shows the funnel plot for one-year mortality for allogeneic transplants. In the funnel plot of Fig. 2, each dot represents a centre. The *x*-axis represents the sample size. In the absence of case mix differences and complete one-year follow-up this would exactly be the number of allogeneic transplants performed in that centre over the benchmarking period. However, the survival model was adjusted for case mix so that it now represents an approximation of the number of allogeneic transplants (van Zwet et al., unpublished). Larger centres are more to the right, smaller ones more to the left. The *y*-axis is observed/expected; this represents the excess events (deaths within one year in this context). A value of two means that in the benchmarking period the number of events is twice as high as expected. Of course, a ratio of observed/expected of two provides more evidence of the centre not performing according to benchmark if that ratio is based on a larger sample size. The funnel shaped curves represent boundaries for significance of the null hypothesis (the centre having the same performance in terms of one-year mortality as the benchmark). The funnels are wider towards the left and narrower towards the right, reflecting the fact that for small centres only very large observed/expected ratios are evidence of underperformance, while large centres need a smaller observed/expected ratio to provide evidence of underperformance. Centres within the inner funnel are performing within range; centres between the inner and outer funnels are performing worse (upper half) or better (lower half) than average, according to the 5% significance level. Finally, centres outside the outer funnel are performing worse (upper half) or better (lower half) than average, according to a multiple-testing adjusted significance level of 5%; if all centres were performing according to benchmark, then there would be a 5% probability that any of the centres were outside the outer funnel.

### One-year loss to follow-up

**Fig. 3:**
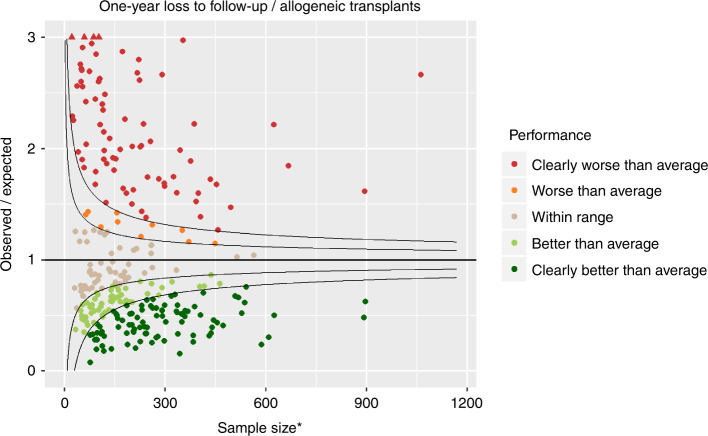
Funnel plot for 1-year loss to follow-up for allogeneic transplants. *Adjusted for patient and centre-specific mortality

Figure 3 shows the funnel plot for one-year follow-up. Clearly the variability in performance is much greater for loss to follow-up, compared to one-year mortality. The funnel plot in Fig. 3 compares the performance of each centre with the benchmark, which is set internally as the average loss to follow-up among all included centres. As such, it is a relative comparison. A more absolute measure of follow-up completeness is provided by the colour coding of the completeness of follow-up. The frequencies were GREEN: 164 (57%), AMBER: 31 (11%), RED: 93 (32%).

### Benchmarking for autologous HSCT

A total of 352 centres, providing data of 65,347 patients have contributed to this benchmarking project for autologous transplants.

**Fig. 4:**
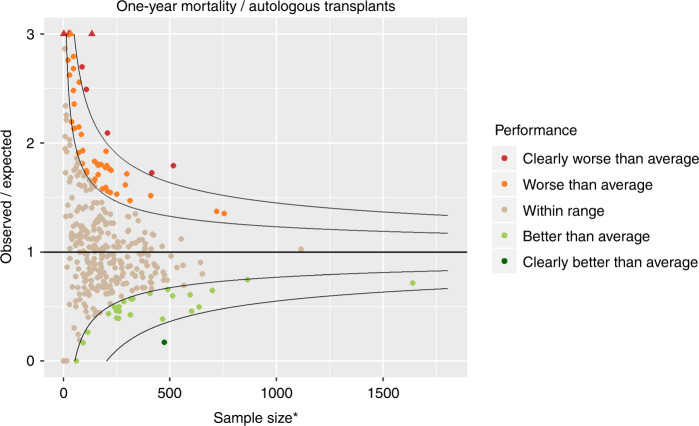
Funnel plot for 1-year mortality following autologous transplantation comparing observed over expected mortality adjusted for case mix and centre follow-up. Results are highly affected by quality of follow-up over the period. *Adjusted for case mix and centre follow up

**Fig. 5:**
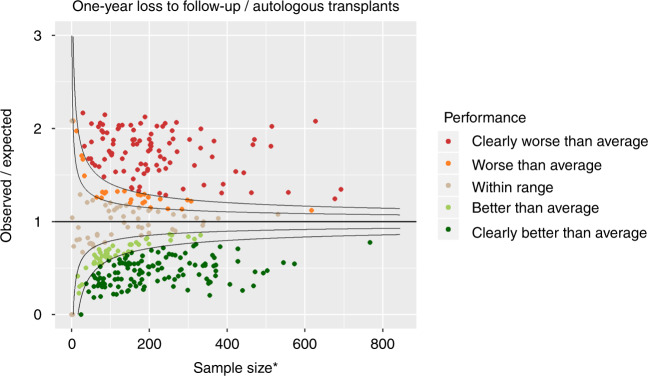
Funnel plot for 1-year loss to follow-up for autologous transplants. *Adjusted for patient and centre-specific mortality

Figures 4 and 5 show the funnel plots for one-year mortality and one-year loss to follow-up, respectively, for the autologous transplants. They were produced in the same way as those for allogeneic transplants, but with a different case mix model.

The colour coding of the completeness of follow-up resulted in GREEN: 155 (44%), AMBER: 38 (11%), RED: 159 (45%).

## Discussion

We have developed a ‘first phase’ of an international registry-based risk-adapted benchmarking system for HSCT outcomes within EBMT, using experience of established national systems and methodology achievable within the EBMT registry. The system is designed to be sustainable through future changes to the underlying technology that is used for the EBMT registry. As a starting point, we appraised several established national systems, both within and outside EBMT. Although nationally accepted, the current systems are not generally consistent or comparable and have limitations with respect to broader application across all EBMT members reporting to the registry. Accommodation of case mix was variable between established systems and generally limited. This meant no single system currently in use in EU countries could be easily rolled out across EBMT. However, we were able to use the analysis to inform the EBMT model.

How benchmarking in HSCT is implemented and sustained across the many EBMT member nations and their HSCT units will be seen over forthcoming years. The uptake may take impetus from the need of centres to fulfil FACT-JACIE 7th edition and subsequent standards, which are likely to further reinforce the value of benchmarking. The aim of the EBMT is to offer peer support through JACIE with a fair, transparent and supportive system to guide centres on possible corrective action to address any underperformance observed through the benchmarking mechanism. Centre anonymity needs to be maintained at this stage of benchmarking development, but ultimately EBMT and JACIE need to be prepared to respond in future in case (or when) centre identification is requested by stakeholders, as is the case for the CIBMTR system where benchmarking outcomes are publicly available. National societies, health services and regulators will also be key to these aspects.

How do we move forward from this ‘first phase’? The immediate objectives are to deliver a ‘first phase’ performance benchmark report to all EBMT member principal investigators to find the position of their centre compared to other anonymised EBMT centres for completeness of follow-up. This is a ‘road test’ for the next and more critical benchmark of survival outcomes. Following assurance of data completeness, we plan to provide reports on 1-year survival outcomes with the proviso that the system is a work-in-progress to those centres that request it, e.g. for preparation of JACIE. Indeed, the model was constrained by the quality of baseline and 1-year follow-up data within the current database. By selecting the top 50% of centres with most complete follow-up for the 1-year survival benchmark, we hope to drive centres to improve follow-up reporting. Benchmarking of follow-up provides a measurable outcome for improvements in resourcing, clinical supervision and education of data managers. When sustainable oversight arrangements for governance have been resourced, 1-year survival outcome performance indicators will be made available to all centres as part of their respective JACIE accreditation processes.

Concurrently, we plan further validation of the model relating to DRI in autologous HSCT, and further detail relating to histocompatibility and other risk stratification factors. Despite the desire of clinical expertise in the group to include as many disease and patient-related variables as possible that are key to transplant outcome, for example, the lack of complete HLA-typing and genetic data meant they had to be excluded from this first phase model. Prospective integration of the benchmarking process with the MACRO registry via the mandatory core dataset will improve completeness of data reporting and form the basis for ongoing development and sustainability of the system as an integral part of the EBMT registry available to all member centres. Ultimately, the MACRO database will be the sustainable source of data, with mandatory reporting of follow-up core data. As our understanding of prognostic data and other risk factors improve, e.g. with the wider routine use and further development of molecular genetics in prognosis and HLA-matching, data collection systems in the registry will accommodate the hierarchy of donor selection according to disease risk [20, 21, 24, 25]. Transplant indications and techniques also change with time, and some indications will be of sufficient size to examine specifically within individual benchmarking exercises, leading to improved quality and scientific insights simultaneously [24, 26, 27]. This may expand to data reporting in non-transplant treatments, including CAR-T cells and other immune effector cells [28]. Importantly, however, whilst we will plan to incorporate additional clinical variables to fine tune risk-adaptation within the expected capabilities of the evolving EBMT registry, this should not compensate for less than ideal pre-transplant decision making, e.g. with poor patient or graft selection, or failure to recognise some transplant modalities as investigational rather than established procedures.

The scientific potential of benchmarking also needs to be developed from a health economic perspective. Firstly, it is fair to recognise that some countries in the EBMT community do not have the same resources as others, acknowledging that macroeconomic factors have been shown to have an impact on HSCT outcomes [29]. This will be even more relevant as centres in low-income and middle-income countries (LMIC) engage with FACT and JACIE initiatives [30]. It may be possible to carefully adjust benchmarking according to health economic indices. Secondly, the impact of benchmarking on patient survival and other outcomes should be continually evaluated to justify the necessary resource utilisation, not just by the EBMT and JACIE, but also by all participating centres, data registries, national societies and health service payers. For long-term sustainability, there is a need for evidence of sufficient impact on patient outcomes to justify the considerable time and resources invested. This is an ‘improvement science’ issue, similar to the exercises performed to demonstrate the potential benefits of implementation of JACIE accreditation [14, 15, 29].

Sustainability also requires dynamic organisational and business planning to maximise acceptance across various national HSCT communities and run a system that can withstand questions and even appeals from centres. A decision was made by the EBMT Board to fund the benchmarking out of increased JACIE fees. This is a practical solution, but has limitations given the variable uptake of JACIE, especially if the goal is quality improvement across all EBMT members. Other potential sources include EBMT membership fees; particularly as full EBMT membership comes with a duty to report data to the registry. All such operational aspects require ongoing review and governance of the benchmarking system at EBMT Board and JACIE committee levels, actioned via an appropriately constituted and resourced oversight committee, which will initially be comprised of clinical members of the COG experienced in delivering benchmarking at the national level and statistical experts, with terms of reference and operational policies which aim to support the EBMT membership generally and at an individual centre level.

In summary, we have delivered the ‘first phase’ of a development for the EBMT. Importantly, benchmarking is being led by the community and not by external stakeholders who may not understand the complexities and progress in HSCT. Inevitably there will be centres that will have some below average results and require explanations. There will also be some high performing centres from which the EBMT community may learn. This is ‘work in progress’ and the system will progressively benefit from engagement and feedback. This ‘first phase’ should not immediately replace national initiatives but complement them aiming eventually to become the preferred option for fair, user-friendly benchmarking throughout EBMT for the benefit of professionals and patients.

## Supplementary information

Position paper Supplementary Information
