# Supplementary material for: Benchmarking of survival outcomes following haematopoietic stem cell transplantation: A review of existing processes and the introduction of an international system from the European Society for Blood and Marrow Transplantation (EBMT) and the Joint Accreditation Committee of ISCT and EBMT (JACIE)
Source: Bone Marrow Transplant. 2019 Oct 21;55(4):681–94. doi: 10.1038/s41409-019-0718-7 (PMC7113189; doi:10.1038/s41409-019-0718-7)
Supplement: Supplementary file 1 — Position paper Supplementary Information [file 41409_2019_718_MOESM1_ESM.docx]

**SUPPLEMENTARY INFORMATION**

### **PART 1. SUMMARIES OF ESTABLISHED BENCHMARKING SYSTEMS**

**PART 2. CLINICAL OUTCOMES GROUP (COG) PROCESSESS, MEMBERSHIP AND ROLES**

### **1. SUMMARIES OF ESTABLISHED BENCHMARKING SYSTEMS**

### **1.1 BENCHMARKING SYSTEMS WITHIN EBMT**

### **1.1.1 United Kingdom: The British Society of Blood and Marrow Transplantation (BSBMT)**

The BSBMT was founded in 1995 and the data registry established in 2001 as a central repository of transplant activity performed by UK and some Republic of Ireland transplant centres using data from the EBMT MED A forms. By 2018 more than 60,000 registrations are held on the database with 52 transplant centres contributing data. Since 2011 the registry has prepared survival curves based on a 6-year rolling time period, the first period being 2002-2007. Overall survival curves are produced with several sub-group categories based on type of transplant (autologous, allogeneic), disease indication, disease stage, conditioning regimen and other parameters. Each transplant centre receives their own overall survival curve estimates with confidence intervals, compared with the estimates and confidence intervals for the remainder of BSBMT centres. The most recent period of reporting was for transplants performed between 2010-15.

Survival curves for individual centres are shared with the UK National Health Service (NHS) who commission and pay for HSCT at a national level, but they are not in the public domain. A grading system has been proposed and introduced based on the relationship of the confidence limits of the overall survival results for a centre compared with the national average with 1 ‘above average’, 2 ‘high average’, 3 ‘average’, 4 ‘low average’ and 5 ‘below average’. An important limitation is the data are not risk- or case mix adjusted, which has limited the scope of the benchmarking process in the UK. An example of BSBMT survival curves are shown in figure A1:


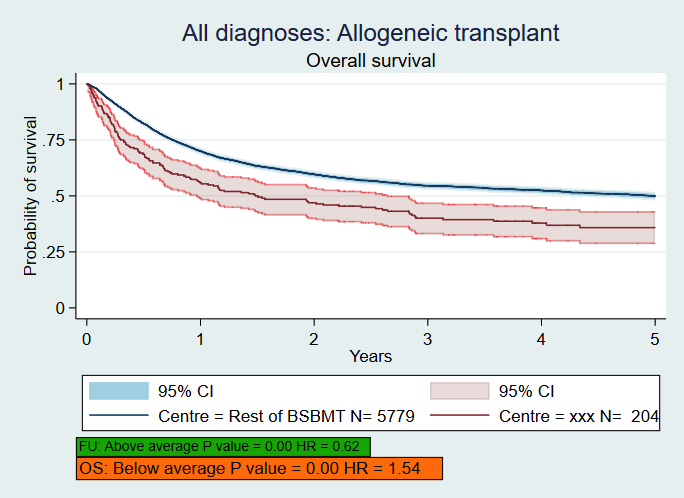


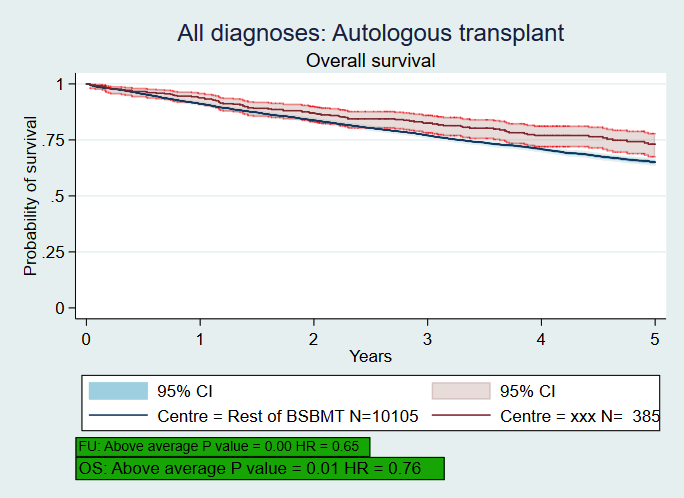


Figures A1a, A1b. Benchmarking of allogenic and autologous HSCT for adult patients (all diagnoses), within the BSBMT exercise, showing examples of underperformance (for allogeneic, A1a) and overperformance (for autologous, A1b).

### **1.1.2 Italy: Gruppo Italiano Trapianto di Midollo Osseo (GITMO) quality assessment of allogeneic transplant activity in adult recipients with Acute Myeloid Leukaemia 2008-2012**

GITMO was created in 1987 as a Scientific Society but has also provided a governance framework for the transplant network, including accreditation criteria mainly based on activity and data reporting. For this reason, the National Competent authority (CNT: National Centre for Transplant) appointed GITMO for data retrieval. Data on HSCT is entered by each transplant program (TP) into the EBMT database with GITMO regularly performing data quality checks and transmitting them to the CNT. Through its clinical trial office, GITMO performs retrospective and prospective studies based on registry data.

The CNT performed the following analysis with GITMO patients with acute myeloid leukaemia (AML) who underwent allogeneic HSCT between 2008 and 2012. An outline analysis was performed to compare the performance of each Italian TP considering the different case mix of patients. The methodology was developed based on the guidelines published by the EBMT (1). The analysis included 2261 transplants performed by 64 Italian transplant programs in the interval 2008-2012. A national estimate was provided for OS, NRM and relapse incidence (RI), (the last two considered as competing risks). The clinical variables considered were: Patient Age; Donor Type, Disease Phase; AML & ALL Transplant Number; Karnofsky status; Donor-Patient Cytomegalovirus Match; Conditioning Myeloablative Regimen; Donor-Patient Sex/Gender Match; Diagnosis to Transplant time interval. The distribution of these variables in each TP was then highlighted to correct the result of each TP according to the complexity of the series (case mix). To accommodate a case mix of more complex and frail patients, TP case series complexity higher or lower compared to the national median. Completeness of follow-up was reported through coloured alerts. Multivariate regression models were used to estimate the risk (hazard) of variables affecting the transplant outcome in terms of OS, Relapse and NRM according to Cox model (for OS) and competing risk analysis with Fine and Gray (2).

The final benchmark was an "adjusted" comparison of the outcome of the different TP based on case mix. Two main indicators were used: 1. The Risk Activity Index of the Centre, which highlights the overall risk performed by the centre based on the comparison of the different case mix of single transplant centre patients; 2. The Estimated Centre Effect based on the multivariate analysis of Cox or competing risks regression (1–3) by a sub distribution hazard regression model. The centre effect was calculated from the multivariate national model. For OS this model is applied to each transplant performed by the centre to calculate the expected number of events. For competing risks regression for each transplant, the sub distribution hazard was utilized to calculate the expected number of events. Comparison between the observed and expected events estimated the centre effect as the percentage deviation of the observed events compared to the expected estimates through its regression model, with values above zero indicating the number of observed events as higher than expected, and vice versa for values below zero. The centre effect therefore implicitly provides an "adjusted" result indicator for the comparison between centres. A new regulation is due to be approved by the Italian Ministry of Health where benchmarking of HSCT outcomes will be a mandatory minimum requirement.

### **1.1.3 Belgium: Belgian Transplant Registry (BTR)**

**Methodology**

The Belgian Transplant registry has performed the following benchmarking analyses using methods very similar to those used by the CIBMTR, except for ethnicity and the Sorror co-morbidity index, which could not be used because data is missing systematically in some centres. Selection criteria for benchmarking include 1) Centre activity of at least 5 transplants per year on average in the population studied (otherwise statistics not reliable), 2) Transplants between 2007 and 2013; 3) Type of transplants: all, including any type of donor, any stem cell source, any number of transplant (1^st^-2^nd^-3^rd^…) and 4) Disease: all except ‘Solid tumour (not Breast)’ and ‘Breast cancer’ as reported in the EBMT database variable ‘Diagnosis’.

For allogeneic HSCT the case mix variables were selected (after a backward elimination procedure) from: Age , CMV status, CMV matching, Conditioning, Disease risk index (adapted from Armand, extended to non-malignant disease), Performance status (Karnofsky), Stem cell source, Sex, Sex matching, Time from diagnosis, Transplant number, Transplant year and HLA matching. This is more comprehensive than in the EBMT Registry, particularly in relation to matching of HLA typing. The EBMT risk score is only tested in univariate analyses but not used for multivariate analysis and benchmarking. For autologous HSCT the case mix variables were selected (by the same procedure) from: Age, Disease risk index (Armand), Performance status (Karnofsky), Sex, Time from diagnosis, Transplant number and Transplant year. EBMT risk score was only tested in univariate analyses but is not used for multivariate analysis and benchmarking. In addition, 3 variables are used in further analysing centres: centre performing only autologous (or only allogeneic) vs auto + allo; time from JACIE accreditation to transplant (all centres now accredited); centre activity volume. This has been summarised in a recent publication from the Belgian Registry for autologous HSCT [26] (manuscript in preparation for allogeneic HSCT).

In terms of statistical methodology, survival was directly obtained from the database of Belgian citizens, so that loss of FU was not an issue. For the small number of non-Belgian citizens, centres are responsible for providing the most recent follow-up. The data extracted from the EBMT database are reviewed for completeness and consistency and centres interrogated for missing or non-consistent data. For instance, cytogenetics in AML and MDS cases were retrieved and interpreted according to the European Leukaemia Network (ELN) criteria. In some instances, data were collected on site.

Outcomes include 1-year and 3-year overall survival and 100-day, 1-year and 3-year TRM and analysis is based on transplant, not the individual patient. Hence, each transplant is included, with follow-up time censored on day of next transplant. In this way, there is accommodation of potentially more difficult transplants and the potential impact of first transplant outcome of second or later transplants (which may be auto vs allo, different modalities, different centres etc). Subgroup analysis includes 1) All allo 2) All allo in adults (>16 years) 3) All unrelated donor transplants in adults (>16 years) 4) All auto 5) All auto in adults (>16 years). The same statistical methodology as the CIBMTR is used (www.[cibmtr.org](http://www.cibmtr.org/ReferenceCenter/SlidesReports/Documents/CIBMTR%20HCT%20Center%20Survival%20Report%20Methodology-FINAL%202017-12-11.pdf)). Survival, adjusted for relevant patient, disease and HCT-related variables, was calculated using a censored data generalized linear regression model that allows inclusion of recipients with incomplete follow-up. The fixed-effect logistic regression model provides information about how the recipients treated in a centre would have fared had they been transplanted at a “generic” transplant centre. This model assumes no centre effect. Centres whose actual survival is below the 95% confidence limits for predicted survival (on the left in Forest plots) are considered underperforming, those within the confidence limits performing as expected and those above (on the right in Forest plots) overperforming. TRM has only been evaluated from an unadjusted point of view by using cumulative incidence.

Results are summarised in figures A3-A5, with funnel and Forest plots for 1-year and 3-year unadjusted survival (fig A3) and adjusted survival incorporating univariate analysis, multivariate model building, and indirect standardization for centre benchmarking (fig A4). Indications for transplantation in each centre compared to indications as published by EBMT in 2010 and 2015 (standard & optional treatments vs in development or not recommended) were analysed and results presented as a funnel plot (fig A5).

Data completeness was also assessed for each variable for each centre.


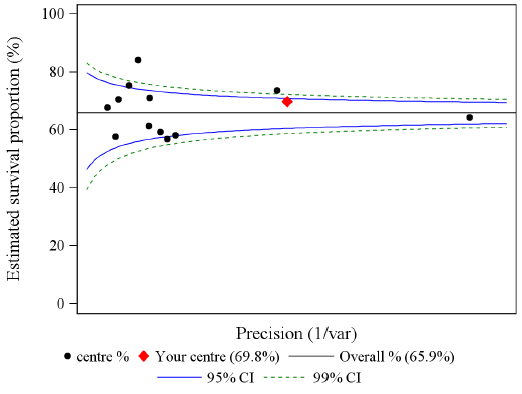


**Figure A3 BTR 1-year and 3-year unadjusted survival: funnel plot**


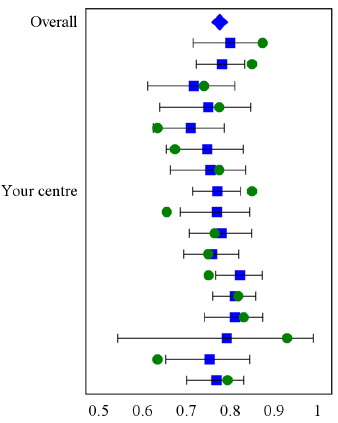


**Figure A4 BTR 1 -year and 3-year adjusted survival: univariate analysis, multivariate model building, indirect standardization for centre benchmarking**


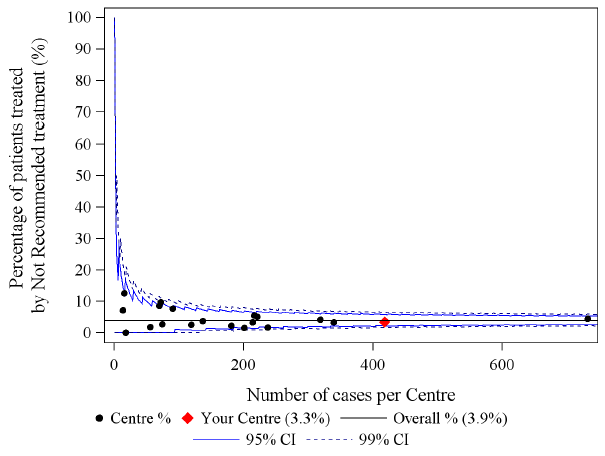


**Figure A5 Standard & optional treatments vs in development or not recommended**

### **
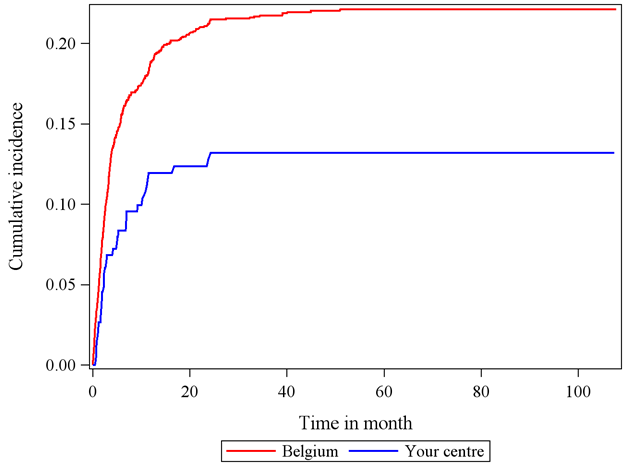
**

**Figure A6 example cumulative incidence of one center and that of Belgium**

### **1.1.4 Germany: The German Stem Cell Transplant Registry (DRST)**

The German Stem Cell Transplant Registry (DRST) was founded in 1998 with the goal of fostering the quality of stem cell transplantation in Germany through 1) documentation and evaluation of HSCT across Germany; 2) provision of data to authorized users of the registry; 3) supporting national and international research.

The DRST today includes 59-member centres performing allogeneic and/ or autologous HSCT across Germany. The registry data has been used to provide annual Benchmarking since 2012 (for transplants done up to 2011). At present, benchmarking is performed for adult transplant centres only. Centre-specific reports are sent annually to the respective head of the transplant centre. Only the DRST and the transplant centre (head) have access to centre-specific reports.

Statistical analysis for the benchmark and the individual centre is performed on a “complete-case” basis. The benchmark includes complete data from all centres. Individual centre results are only presented when a minimum of 20 events were recorded. Results include tables displaying transplant numbers reported to the registry (as reflected in the EBMT database) and the completeness of follow-up. Outcome results are displayed in form of survival curves (overall survival) and cumulative incidence of competing risks (non-relapse mortality) including transplants from the preceding 10 years.

Risk stratification is not performed. Results are however presented for all first allogeneic transplants separated according to DRST-EBMT risk groups (example is shown in figure below). The DRST-EBMT risk score is an age-adjusted EBMT-score (Table A1). Furthermore, results are presented separately for disease entity (e.g. AML, ALL, CML, NHL) and in case of AML and ALL as 1^st^ CR or beyond 1^st^ CR, for CML as 1^st^ CP or beyond 1^st^ CP.

In a last part, data on deaths and causes of death (relapse, TRM, second malignancy, other) are presented in tables. Day 100 mortality for all transplants is presented for the preceding 5 years (e.g. 2012 -2016) for all of Germany as well as the specific centre.

**Figure A2 Overall survival according to the DRST system adjusted by the DRST_EBMT risk score (table below).**

| **Risk factor** | **Score** |
| --- | --- |
| **Patient age** | |
| ≤ 30 | 0 |
| 31-45 | 1 |
| 46-60 | 2 |
| >60 | 3 |
| **Disease Stage** | |
| Early | 0 |
| Intermediate | 1 |
| advanced | 2 |
| **Interval from diagnosis to transplant** | |
| < 12 months | 0 |
| ≥ 12 months | 1 |
| **Donor type** | |
| HLA-identical sibling | 0 |
| Other | 1 |

**Table A1 (needs caption)**

### **1.1.5 France:** Agence de la biomédecine **(ABM) & SFGM-TC (Société Francophone de Greffe de Moelle et de Thérapie Cellulaire)**

Allogeneic HSCT activity is supervised by a government agency: Agence de la biomédecine (ABM) established in 2004 (for further details on ABM see: [www.agence-biomedecine.fr](http://www.agence-biomedecine.fr)). Each authorized French team must report allo HSCT activity yearly through a report form generated by ABM. Data on this form are further cross-checked with data in the EBMT database in close relationship with SFGM-TC (Francophone Society of Bone Marrow Transplantation and Cellular Therapy) data manager before extraction to produce a public annual report.

One of the major missions of ABM is to conduct evaluation of allogeneic HSCT results as well as of solid organ transplantation results. There is no current evaluation of autologous HSCT. The first evaluation, released in November 2010, focused on allogeneic HSCT performed from 2001 to 2006. It was presented at a poster session at EBMT meeting, Paris 2011 (4). A second evaluation based on allogeneic HSCT performed from 2007 to 2011 was released in 2014, and a third evaluation is scheduled for November 2019 covering allogeneic HSCT performed from 2012 to 2017.

End points are 1-year Overall Survival (OS) and Transplant Related Mortality (TRM). For each period of evaluation, the population considered is represented by any first allograft. Each transplant team is contacted and requested to complete extensively both clinical parameters and 1-year survival (less than 10% missing data for the last evaluation). Data are then extracted from the EBMT database. The HLA typing data are specifically cross-checked with data extracted from French Registry of unrelated stem cell donors (RFGM) allowing for a complete and precise HLA matching.

There are some exclusion criteria: Teams performing less than 25 allogeneic HSCT during the five-year evaluation period, rare diagnosis (i.e. solid tumours and other diagnosis accounting for less than 1% of indications, and haploidentical as well as syngeneic transplants). In order to take into account case mix, the following items are retrieved: Type and status of disease, age of recipient, sex of donor and recipient and sex-matching, HLA matching, cell source (BM, PBSC, CB), CMV serostatus of donor and recipient and CMV status matching, conditioning regimen (MA or NMA as to Bacigalupo et al, BBMT 2009(5)), previous autologous HSCT and year of transplant.

For the purpose of statistical analysis, the first step is to define prognostic factors impacting 1-year OS and TRM in overall national cohort, through univariate (Kaplan-Meier) and multivariate (Cox) analysis for OS and Fine and Gray for TRM.

On the basis of the multivariate model, the next step is to compute for each team: 1) the 1 year expected TRM or overall mortality rate, given the patients case mix of the team 2) the 1 year observed TRM or overall mortality rate 3) the 1 year adjusted TRM or overall mortality rate computed as the national 1 year TRM or overall mortality rate weighted by the ratio between observed and expected team-specific mortality rate. The final step plots each team on a “funnel plot” showing 1 year national TRM or overall mortality rate with 99% confidence interval calculated according to the size of the team(6). Each plotted dot represents the 1-year adjusted TRM or OS of one team (figure A6). Separate analysis is produced for adults and children. A dot, representing a team outside of the funnel plot, means either better or worse result for this team as compared to the national norm. Each team receives the figure with its position indicated but cannot see nor be seen by the other teams. In case of being outside of the funnel plot, the team is contacted by ABM to understand the reasons, with the aim of improving outcomes.

**Figure A6. 1-year adjusted TRM. Each dot = one team. Horizontal line= TRM national rate. Red line= 99% upper CI. Green line=99% lower CI. X axis= number of transplants performed during the evaluation period.**

### **1.1.6 Switzerland; the Swiss Blood Stem Cell Transplantation (SBST)**

In order to control for the proliferation of centres doing highly specialized medicine, the Swiss authorities have created a body termed Intercantonal coordination of highly specialized medicine. This body has determined that allogeneic and paediatric autologous HCT is to be under the auspices of this body but not autologous HCT in adults. Swiss transplant centres are qualified through SBST, a national working group with representatives of each centre, functioning as a consultative body to the Swiss national donor registry (SBSC) and as an HCT working group of the Swiss Society of Haematology. This qualification includes verification of data sharing and JACIE accreditation status and underpinned by the Swiss law, which stipulates accreditation and such qualification as a necessary requirement for permission to operate as a transplant centre and to bill to public health insurances. The health ministry has established a list of accepted transplant indications and qualifies indications as standard or as acceptable only in the framework of clinical trials. Currently there are 4 allogeneic centres in 3 locations in Switzerland (Basel, Geneva, Zurich, Zurich having a separate adult and dedicated paediatric centre) and 9 centres doing autologous HCT (the number for 2017 were 252 patients with allogeneic and 668 with autologous HCT). Therefore, SBST reports annually to the authorities including indications and outcomes such as survival, progression fee survival, non-relapse mortality and relapse incidence for allogeneic and autologous HCT and will extend this to other cellular therapies in the coming years.

Since 1997, all autologous and allogeneic HSCT procedures are fully recorded and reported to EBMT. SBST operates a consenting procedure for transplant indications outside the lists accepted by the health ministry and for activating donor searches in older patients where the indication and comorbidity-indices are reported prior to activating unrelated donor search. This consenting procedure functions by circular email consultation and finds an answer generally within 48 hours.

SBST organizes an annual meeting where the number and indications for HCT of the preceding years are discussed and outcomes are evaluated. Although this does not correspond to benchmarking system in the proper sense, data are shown and discussed in a transparent way including display of outcome by transplant centre. In addition, periodically analysed data are published, mostly in national journals (e.g. Passweg J, Baldomero H, Stern M, Bargetzi M, Ghielmini M, Leibundgut K, et al. Hematopoietic stem cell transplantation in Switzerland: a comprehensive quality control report on centre effect. Swiss Med Wkly [Internet]. 2010 Jun 12;140(23–24):326–34.).

### **1.1.7 Spain: Spanish Society for Hematopoietic Transplantation and Cell Therapy (GETH), National Transplant Organization (ONT), Spanish Society of Haematology (SEHH)**

In 2017 the Spanish Society for Hematopoietic Transplantation and Cell Therapy (GETH) launched a project to improve quality processes and public access to outcome data in the field of HSCT. Participation was voluntary and open to all HSCT programs across the country. Data source was the transplant information reported to the EBMT Registry, restricted in the initial phase to transplants performed in adult HSCT teams on patients 15 years-old or older at the time of transplant. The analysis was conceived in several steps: 1) to analyze the representativeness of the HSCT reported to the EBMT in relation to the official activity records in Spain, 2) to monitor data quality, and 3) to report general unadjusted preliminary outcomes in terms of overall survival 1 year after transplant.

Results of our initial analysis for 2014 and 2015, with data downloaded from ProMISe on 15 February 2017, showed that 41 out of 71 (58%) Spanish HSCT teams were EBMT members, representing 83% of the Spanish HSCT activity in study period. Nevertheless, only 3.550 of the 4.612 (77%) transplants performed in EBMT centers had been reported in the EBMT Registry. Thus, the transplant information included in ProMISe for years 2014 and 2015 represented the 64% of the total national activity in Spain. Overall survival for this initial period, including only patients with at least 1-year follow-up, was 83%, 58% and 49% in allogenic HSCT and 97%, 85% and 80% in autologous HSCT at 3, 6 and 12 months after HSCT, respectively.

In 2019, a new analysis shows that HSCT activity reported to the EBMT Registry increased to 67% of the overall activity in Spain for the period 2016-2017. To increase the quality of the data, an additional proposal has been made that HSCT data would be included in this broad national analysis only from centers that report >95% of their transplant activity and >90% of a set of core data fields including diagnosis, type of transplant, donor type, chronologic number of transplant and status at 1 year.

From these preliminary analyses, and taking into account the progress of the EBMT benchmarking initiative, the next step and strong commitment of GETH, in collaboration with the Spanish Hematology Society (SEHH) and the National Transplant Organization (ONT) will be working together to improve data reporting to the Registry and data quality from Spanish HSCT centers, in order to join the EBMT benchmark project and take advantage of the methodological strength of this initiative.

### **1.2 BENCHMARKING SYSTEMS OUTSIDE OF EBMT**

**1.2.1 USA: United States Centre Specific Survival Reporting Experience**

Centre-specific risk-adjusted survival comparisons have been a requirement of the US government backed hematopoietic stem cell transplant program since 1994. Initially, the 1990 Transplant Amendments Act required such reporting for U.S. unrelated donor transplants; the Stem Cell Therapeutic and Research Act of 2005 (re-authorized in 2010 and 2015) added similar requirements for related HCT. The Centre for International Blood and Marrow Transplant Research (CIBMTR) a research collaboration between the NMDP and Medical College of Wisconsin has been responsible for producing the report as the contract holder for the Stem Cell Therapeutic Outcome Database (SCTOD) since 2007. The purpose of the report is to provide potential allogeneic HSCT recipients, their families and the public with a comparison of survival rates among the centres in the C.W. Bill Young Cell Transplantation Program network. Centre performance is publicly available through an online [Transplant Centre directory](https://bethematch.org/tcdirectory/search) and transplant centres may use these reports for quality improvement initiatives.

The current methodology was implemented in 2005 and allows for direct comparisons over a decade of performance evaluations. One-year overall survival was chosen as the reported outcome because it is an unambiguous endpoint that balances HSCT centre control, type of transplant regimen and the preferred outcome (long-term survival) desired by the patient and society. The analysis includes all first related or unrelated donor HHSCT performed during a 3-year time interval with one-year minimum follow-up. Every effort is made to ensure completeness of follow-up and accuracy of the data for each recipient included in the model using standard operating procedures for data checks and review.

Occasionally, recipients are indeed lost to follow-up, and final survival status at one year is unknown. To address this problem, the analysis only includes centres where the one-year survival status is known for at least 90% of their transplanted recipients. One-year survival, adjusted for relevant patient, disease and HCT-related variables, is calculated using a censored data logistic regression model the fixed-effect logistic regression model provides information about how the recipients treated in a centre would have fared had they been transplanted at a “generic” transplant centre. This model assumes no centre effect. Specifics of the modelling procedure can be found at [cibmtr.org](https://www.cibmtr.org/ReferenceCenter/SlidesReports/USStats/Documents/CIBMTR_HCT_Center_Survival_Report_Methodology.pdf). Centres whose actual survival is below the 95% confidence limits for predicted survival are considered underperforming, those within the confidence limits performing as expected and those above over performing.

The CIBMTR engages the broad HSCT community bi-annually, through the Centre Outcomes Forum, to continually revise and improve the processes and methodologies used for analysis and reporting. Participants include HCT physicians (U.S. and international), biostatisticians, government funding agencies, the solid organ transplant community, patients, private and public payers, and experts in hospital and quality outcomes reporting. Topics include review of the statistical methodology, revisions of variables collected for risk adjustment, public dissemination of results, and utilization for performance improvement. Proceedings from past forums are available online ([link](http://www.cibmtr.org/Meetings/Materials/CSOAForum/pages/index.aspx)). This transparent process with stakeholders is critical for gaining acceptance, maximizing validity and utility of the centre specific survival reporting efforts. The CIBMTR continually updates the analysis approach to stay relevant with the rapidly evolving HCT field. However, because of the lagging nature of the process, changes in data collection to maintain relevance take several years to appear in the analyses.

### **1.2.2 Australia & New Zealand: Australasian Bone Marrow Transplant Recipient Registry (ABMTRR)**

The ABMTRR was established in 1992 and aims to collect data for all HSCT performed in Australia and New Zealand. It operates under the auspices of the Bone Marrow Transplant Society of Australia and New Zealand (BMTSANZ). There are currently 42 contributing centres in Australia and 6 in New Zealand, with a total of 35,000 autologous and allogeneic transplants in the database and accruing at more than 2,000 transplants per year. Whilst it is expected that all transplants will be recorded, registry reporting is not mandatory. Each year an Annual Data Summary is produced, detailing transplant activity and outcomes for these two countries. The ABMTRR data collection closely aligns with EBMT MED-A, with some additional local items.

The ABMTRR has provided quality and benchmarking data on an ad hoc basis to individual hospitals, state-based health organisations and the Australia New Zealand Children’s Haematology Oncology Group (ANZCHOG) BMT subgroup for their annual HSCT audit. These reports have included survival, transplant related mortality (TRM), graft vs host disease (GVHD) and engraftment for both allogeneic and autologous transplants. The methodology used for the CIBMTR Centre specific Stem Cell Therapeutic Outcome Database has been adapted as possible, although not all these data items are available in the ABMTRR collection, or even when available, may be limited by small numbers. This methodology has not yet been implemented for autologous transplants, although some preliminary analyses have been explored. Comparative Kaplan-Meier survival curves with 95% confidence intervals are provided for centre versus the rest of the database and may be broken down into disease specific or other comparable groups. Cumulative incidence curves are shown for engraftment, GVHD and TRM. These graphical representations are usually well received by contributors; however, it is recognised that they are mostly unadjusted therefore not necessarily reliable for benchmarking purposes.

The online database Australasian Stem Cell Transplant Registry Online (ASTRO) has been in operation since 2013 and is currently being upgraded to include business intelligence software and R capability. The ABMTRR aims to implement systematic annual benchmarking for all centres and eventually have some benchmarking capability available from a dashboard. Whilst working towards a more robust methodology to include all transplants that is feasible in the local setting, we recognise the need for complete and reliable data. There is increasing demand for benchmarking analyses for centres to comply with accreditation standards, therefore it is imperative that this benchmarking is a measure of service quality rather than data quality. Quarterly feedback of data quality indicators to centres has helped to improve the quality of data available for benchmarking, and it is hoped that the database dashboard will further engage contributors to provide this data.

**2. CLINICAL OUTCOMES GROUP (COG) PROCESSESS, MEMBERSHIP AND ROLES**

As an intrinsic part of the EBMT registry upgrade ‘Project 2020’, a work package *“Clinical quality assurance of patient survival outcomes by international benchmarking”* was incorporated. Following initial discussions within meetings of the EBMT Board and EBMT Annual Meeting in Marseille in 2017-18, the Leiden University Medical Centre (LUMC) team was appointed to develop statistical methodology for benchmarking for the EBMT. Subsequently, a multi-national group of senior HSCT clinicians, registry managers, EBMT (including JACIE) staff and biostatisticians from LUMC, EBMT Patient Advocacy Committee and national societies met systematically as the ‘Clinical Outcomes Group’ on a monthly basis from the Lisbon Annual Meeting in March 2018 to June 2019. CIBMTR and Australasian (ABMTRR) representatives were co-opted in order to access their experience. After agreeing Terms of Reference, discussions progressed either by e-mail or teleconference with periodic in-person meetings. Statistical aspects were led by the Department of Biomedical Data Sciences, LUMC with input from the EBMT Statistical Committee and national statistical representatives. Data was sourced from the EBMT registry which is supported by mandatory reporting of anonymised data from routinely consented patients by full EBMT centre members, and supplemented with data from the broader EBMT actvity survey. The introductory phase of the benchmarking system and plans for development were finalised and ratified at the EBMT Board meeting in June 2019.

**Membership and roles included:**

John Snowden, Chair of Clinical Outcomes Group, Chair JACIE, and EBMT Board/Scientific Council member with Portfolio of Registry, Sheffield, UK

Nigel Brook, 2020 Project Manager, EBMT Central Registry Office, London, UK

Eoin McGrath, JACIE Operations Manager, EBMT Executive Office, Barcelona, Spain

Erik van Zwet, Member, Department of Biomedical Data Sciences, Leiden, The Netherlands

Hein Putter, Professor, Department of Biomedical Data Sciences, Leiden, The Netherlands

Ronald Brand, Prof.em. Good Research Data Management (GRDM), Biostatistician, Department of Biomedical Data Sciences, Section Advanced Data Management, Leiden, The Netherlands

Myriam Labopin, Chair of EBMT Statistical Committee, Paris, France

Carmen Ruiz de Elvira, Head of the EBMT Registry, London, UK

Kim Orchard, Chair of JACIE Accreditation Committee

Riccardo Saccardi, JACIE Medical Director, Florence, Italy

Rafael F. Duarte, EBMT Secretary, Spain

Alois Gratwohl, Physician, EBMT Honorary Member, Switzerland

Jakob Passweg & Helen Baldomero, EBMT/WBMT Transplant Activity Survey office Swiss National Transplant Registry SBST, Basel, Switzerland

Elena Oldani, Data Management, Secretary and Italian National BMT Registry – GITMO, Italy

Francesca Bonifazi, Principal Investigator, Secretary and Italian National BMT Registry – GITMO, Italy

Francis Ayuk Ayuketang, Physician, University Hospital Eppendorf

Julia Perry, Head of BSBMT Data Registry, UK

Rachel Pearce, Statistician, BSBMT Data Registry, UK

Noel Milpied, Hématologie clinique et thérapie celllulaire, France

Yves Beguin, Chair of Belgian Transplant Registry, Belgium

Doug Rizzo, Senior Scientific Director, CIBMTR (co-opted)

Steve Spellman, Scientific Director, CIBMTR (co-opted)

John Moore & Leonie Wilcox, Australasian Bone Marrow Transplant Recipient Registry (ABMTRR)

# Per Ljungman, Chair of the Registry Committee, Sweden

**Terms of reference included:**

The clinicians' contribution will be to ensure that the benchmarking system under development (for which the statisticians take responsibility as far as the methodology per se is concerned) is actually useful for EBMT and for JACIE purposes.

They should actively advise the statisticians about the way the system should be documented and explained to the general EBMT community in order to maximize future take-up or acceptance of the system.

The clinicians should be willing to gain a basic understanding of the proposed statistical benchmarking approach (for which the statisticians are of course responsible); be willing to offer their expertise, both on request or proactively, to the statisticians in order to guarantee both the applicability to practice of HSCT in EBMT centres and the interpretability of the ensuing ranking (benchmarking) in view of the complexity of the transplant process, the patient selection processes and the feasibility of a useful interpretation and application of a benchmarking software infrastructure for EBMT.

The expert group will report by the end of 2018 to John Snowden, Chair of Work Package: Clinical quality assurance of patient survival outcomes by international benchmarking, the Board of EBMT and the JACIE Committee on implementation of the mechanism and including identification of any additional development necessary to avoid misuse or misinterpretation of the benchmarking in daily practice.

The statisticians in this project will also communicate directly with the EBMT Statistical Committee members to ensure maximum support from that angle and to make sure the system to be delivered will be firmly rooted in the EBMT organisation.

During 2019 the established methodology will be converted to an IT (software) to be decided (stand-alone or integrated into MACRO). The group of expert clinicians will also advise on the interpretation of the benchmarking information in the development version of the software to be created to ensure that all EBMT members will be able to use and understand the consequences of the benchmarking system.

Moreover, the expert group will advise to what extend the results are plausible and reliable enough to be presented to centres confidentially; to the EBMT as a whole; to satisfy JACIE requirements, etc.

**Confidentiality and conflict of interest**

By accepting this invitation to participate, participants also agree to the non-disclosure of any information they receive about centre-specific data and benchmarks and the discussions concerning these sensitive data, outcome measures and relative performance indicators.

# Reference Documentation

[Concept Paper Registry Update v4](file://C:\Users\jasnowden\AppData\Local\Microsoft\Carmen\AppData\Local\Microsoft\Windows\Temporary%20Internet%20Files\Content.Outlook\AppData\Local\Microsoft\Windows\nkroeger\AppData\Carmen\AppData\Local\Microsoft\Windows\Temporary%20Internet%20Files\Content.Outlook\First%20attempt\2012\Concept_Paper_Registry_Update_v4.pdf).pdf

[Features Functionality.xlsx](file://C:\Users\jasnowden\AppData\Local\Microsoft\Carmen\AppData\Local\Microsoft\Windows\Temporary%20Internet%20Files\Content.Outlook\AppData\Local\Microsoft\Windows\nkroeger\AppData\Carmen\AppData\Local\Microsoft\Windows\Temporary%20Internet%20Files\Content.Outlook\Summary%20of%20requirements\Features%20%20Functionality.xlsx)

[EBMT Detailed Requirements - v2.6.doc](file://C:\Users\jasnowden\AppData\Local\Microsoft\Carmen\AppData\Local\Microsoft\Windows\Temporary%20Internet%20Files\Content.Outlook\AppData\Local\Microsoft\Windows\nkroeger\AppData\Carmen\AppData\Local\Microsoft\Windows\Temporary%20Internet%20Files\Content.Outlook\2014%20November%20snapshot\EBMT%20Detailed%20Requirements%20-%20v2.6.docx)

[EBMT Registry Project 201510 Technical Evaluation Summary EN_F01.xlsx](file://C:\Users\jasnowden\AppData\Local\Microsoft\Carmen\AppData\Local\Microsoft\Windows\Temporary%20Internet%20Files\Content.Outlook\AppData\Local\Microsoft\Windows\nkroeger\AppData\Carmen\AppData\Local\Microsoft\Windows\Temporary%20Internet%20Files\Content.Outlook\2015%20search\EBMT%20Registry%20Project%20%20201510%20Technical%20Evaluation%20Summary%20EN_F01.xlsx)

**CO-AUTHORSHIP**

Co-authorship of the document is from the members of the Clinical Outcomes Group (COG) who actively participated in the development processes, including individuals who provided substantive details of established national benchmarking process and EBMT Board and Scientific Council Members active in the process and/or approving the final manuscript.

Bibliography (Supplementary Information)

1. Iacobelli S. Suggestions on the use of statistical methodologies in studies of the European Group for Blood and Marrow Transplantation. Bone Marrow Transplant. 2013;48(S1):S1–37.

2. Fine JP, Gray RJ. A Proportional Hazards Model for the Subdistribution of a Competing Risk. J Am Stat Assoc. 1999;94(446):496–509.

3. He K, Schaubel DE. Methods for comparing center-specific survival outcomes using direct standardization. Stat Med. 2014;33(12):2048–61.

4. Mesnil F, Shojaei T, Laouabdia K FC. Evaluation of allogeneic HSCT outcomes in 34 French centres. In : Abstracts of the Data Management Group EBMT 2011. Bone Marrow Transplant. 2011;(46):S390–S391.

5. Bacigalupo A, Ballen K, Rizzo D, Giralt S, Lazarus H, Ho V, et al. Defining the Intensity of Conditioning Regimens: Working Definitions. Biol Blood Marrow Transplant. 2009;15(12):1628–33.

6. Spiegelhalter DJ. Funnel plots for comparing institutional performance. Stat Med 2005;24(8):1185–202.
